# Premedication with a cathepsin C inhibitor alleviates early primary graft dysfunction in mouse recipients after lung transplantation

**DOI:** 10.1038/s41598-019-46206-8

**Published:** 2019-07-09

**Authors:** Salome R. T. Rehm, Natalia F. Smirnova, Carmela Morrone, Jessica Götzfried, Annette Feuchtinger, John Pedersen, Brice Korkmaz, Ali Önder Yildirim, Dieter E. Jenne

**Affiliations:** 1 Comprehensive Pneumology Center (CPC-M), Institute of Lung Biology and Disease (iLBD), Helmholtz Zentrum München and University Hospital of the Ludwig-Maximilians University (LMU), Munich, 81377 Germany; 2 Research Unit Analytical Pathology, Institute of Pathology, Helmholtz Zentrum München, Neuherberg, Germany; 3 Neuprozyme Therapeutics and Unizyme Laboratories A/S, Agern Allé, DK2970 Hoersholm, Denmark; 4 INSERM U-1100, “Centre d’Etude des Pathologies Respiratoires (CEPR)”, and Université de Tours, Tours, France; 5 German Center for Lung Research, Munich, 81377 Germany; 6 Max Planck Institute of Neurobiology, Planegg-Martinsried, 82152 Germany

**Keywords:** Respiration, Proteases, Allotransplantation

## Abstract

Neutrophil serine proteases (NSPs), like proteinase 3 (PR3) and neutrophil elastase (NE) are implicated in ischemia-reperfusion responses after lung transplantation (LTx). Cathepsin C (CatC) acts as the key regulator of NSP maturation during biosynthesis. We hypothesized that CatC inhibitors would reduce vascular breakdown and inflammation during reperfusion in pretreated lung transplant recipients by blocking NSP maturation in the bone marrow. An orthotopic LTx model in mice was used to mimic the induction of an ischemia-reperfusion response after 18 h cold storage of the graft and LTx. Recipient mice were treated *subcutaneously* with a chemical CatC inhibitor (ICatC) for 10 days prior to LTx. We examined the effect of the ICatC treatment by measuring the gas exchange function of the left lung graft, protein content, neutrophil numbers and NSP activities in the bone marrow 4 h after reperfusion. Pre-operative ICatC treatment of the recipient mice improved early graft function and lead to the disappearance of active NSP protein in the transplanted lung. NSP activities were also substantially reduced in bone marrow neutrophils. Preemptive NSP reduction by CatC inhibition may prove to be a viable and effective approach to reduce immediate ischemia reperfusion responses after LTx.

## Introduction

Lung transplantation (LTx) is the final treatment option for many patients suffering from end-stage pulmonary disease. A major complication of this thoracic surgical procedure is primary graft dysfunction (PGD), the leading cause of early post-transplantation morbidity and mortality^1–3^. It is caused by ischemia-reperfusion injury, associated with the release of neutrophil serine proteases (NSPs) during reperfusion of the ischemic lung transplant^4–7^. While alpha-1-antitrypsin (AAT) added to the preservation solution protects murine donor lungs during cold storage and initially during first blood passages after reperfusion by its anti-protease activity^8^, circulating neutrophils from the recipient still contain active serine proteases including neutrophil elastase (NE), proteinase (PR3), cathepsin G (CatG) and neutrophil serine protease 4 (NSP4), which are not all inhibited by AAT.

Granule-stored NSPs are initially synthesized as inactive zymogens and are converted by cathepsin C (CatC) into their mature active form during packaging and storage into primary azurophilic granules in promyelocytic precursor cells of the bone marrow^9–11^. CatC, also known as dipeptidylpeptidase I, is a cysteine protease, belongs to the papain superfamily and acts as an N-terminal exopeptidase^12^. CatC removes distinct dipeptides from the flexible N-terminus of proteins at a moderately acidic pH and, in particular, removes pro-peptide sequences from zymogens of NSPs^13^. The latter are granule-stored in their active conformation in immature myeloid cells of the bone marrow. Homozygous loss of function mutations in humans are the genetic cause of the rare Papillon-Lefèvre syndrome (PLS, OMIM: 245000) primarily occurring in consanguineous families^14,15^. Its clinical manifestations are quite variable, but not life-threatening^16,17^.

Pharmacological inhibition of normal CatC in healthy humans has been evaluated in phase 1 clinical trials with two different small molecule inhibitors^18,19^. One drug candidate (AZD7986, identical to INS1007) clearly reduced the NE activity in human blood neutrophils by 60% after repeated dosing 12 days after treatment^18^. A third inhibitor, ICatC (XPZ-01), available to us, was also developed and successfully evaluated in a non-human primate model (Macaca fascicularis). It reduced both the protein levels and activities of NE and PR3 in peripheral blood and bronchoalveolar lavage (BAL) neutrophils attracted to the airway lumen by intratracheal lipopolysaccharide (LPS) instillation^20^. CatC inhibitors are currently considered as promising therapeutics for various neutrophil-driven inflammatory diseases, such as COPD and bronchiectasis^21^.

In this study, we have transplanted the left lung from a donor mouse to an isogenic recipient after 18 h of cold storage to induce a severe ischemia-reperfusion response in the transplant. To preemptively reduce the NSP burden in recipient mice, we applied a CatC inhibitor, ICatC, twice daily for 10 days prior to experimental transplantation. We used a highly cell permeable reversible CatC inhibitor ((S)-2-amino-N-((1S, 2S)-1-cyano-2-(4’-(4-methylpiperazin-1-ylsulfonyl) biphenyl-4-yl)cyclopropyl)butanamide), XPZ-01 (Unizyme Laboratories) which has also been assessed in mice (34) very recently.

We found that 10 days of treatment with ICatC significantly reduced serine protease activity in bone marrow cells of mice and improved gas exchange after subsequent LTx compared to the control group. Moreover, our results indicate that the breakdown of the epithelial lung barrier is reduced by ICatC treatment. Preemptive depletion of serine protease activities in circulating neutrophils and protection against proteolysis of multiple protease targets could improve the clinical outcome of transplanted patients in the future.

## Results

### NSPs are eliminated by pharmacological inhibition of CatC

**Figure 1:**
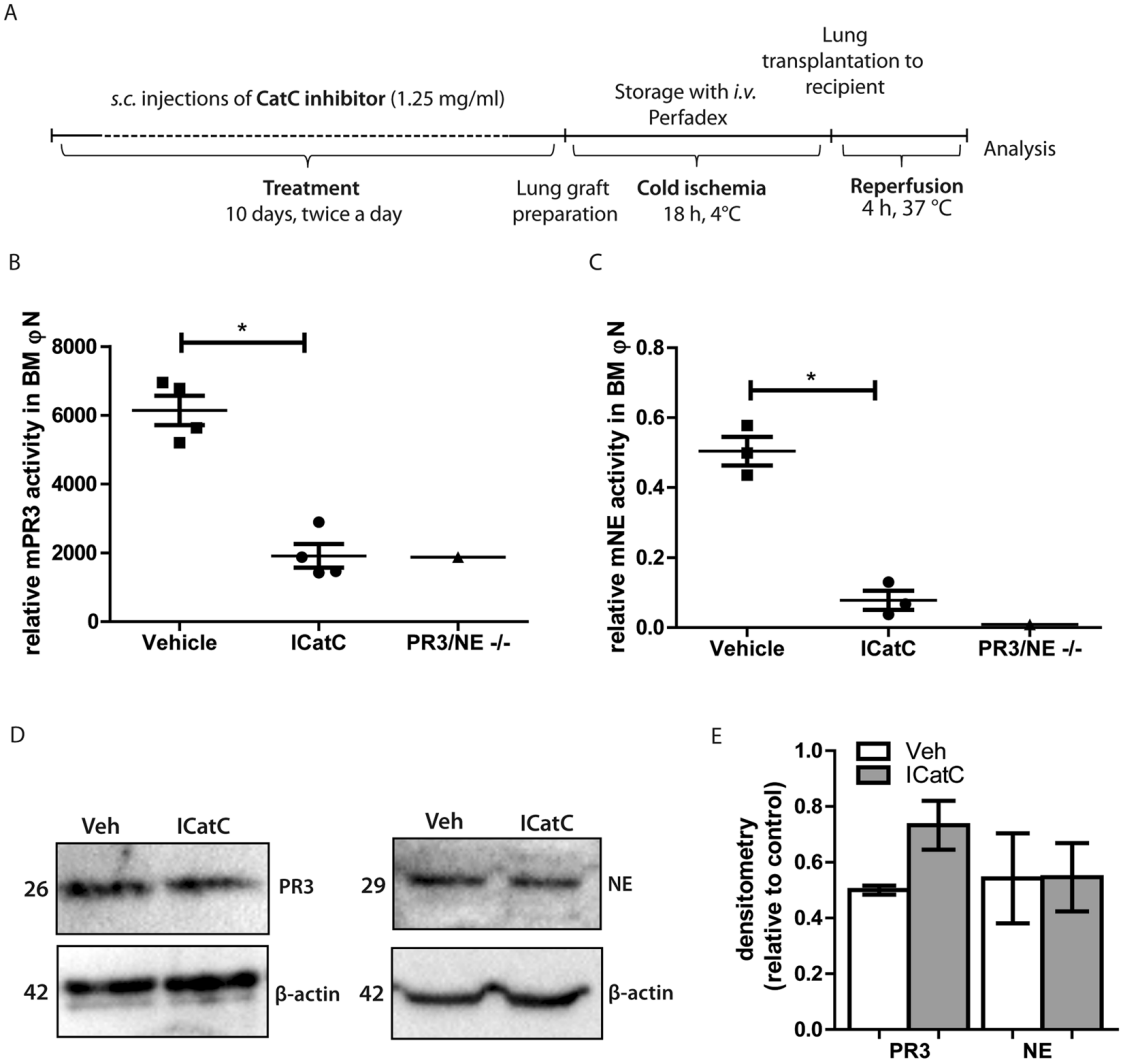
ICatC pre-treatment eliminates serine protease maturation in the bone marrow. (**A**) Experimental setup of preoperative inhibitor application, cold ischemic storage, and orthotopic lung transplantation in C57BL/6 mice. C57BL/6 recipient mice were *subcutaneously* injected with ICatC (1.25 mg/ml) or vehicle twice a day for 10 days. On day 10, donor left lung grafts were perfused with Perfadex and stored in Perfadex at 4 °C for 18 hours (cold ischemia). The left lung was orthotopically transplanted to the recipient and mice were sacrificed for analyses 4 h after reperfusion. (**B**) PR3 and (**C**) NE activity measurements in bone marrow-derived (BM) neutrophil lysates from vehicle and ICatC treated mice using the murine PR3-specific FRET substrate TAMRA-GVRRVVQVQD-Dap-(CF) and ABZ-GAVVASELR-Y-(NO_2_)-D substrate for murine NE at 10 µM to determine the remaining activities. BM derived neutrophil lysates from PR3/NE −/− mice served as negative control. Western blot images (**D**) and densitometry (**E**) of PR3 and NE in PMN lysates after vehicle and ICatC treatment. Immunodetection of β-actin served as a loading control (lower panel). Full western blots are provided in supplementary information 1A,B. Statistical significance between vehicle and ICatC treatment is indicated with *horizontal brackets* and *asterisks*. Values are represented as means ± SEM of 3/4 mice per group and compared with a Mann-Whitney test (*p ≤ 0.05).

Waiting times of the patient for LTx could be used to improve the outcome of lung transplant surgery by pre-operative application of CatC inhibitors. To evaluate the potential beneficial effects of CatC inhibitors in pre-treated recipients, we have chosen a murine experimental model of orthotopic LTx outlined and illustrated in Fig. 1A. From a previous study with rats^22^ we knew that a pretreatment period of 8 to 10 days should be sufficient to reduce the serine protease activities of the circulating neutrophil pool and to eventually replace them by newly formed protease-depleted neutrophils. Because of the relatively short half-life of ICatC, twice daily dosing was chosen to reach steady state levels after 10 days (Fig. 1A).

To ascertain that prolonged ICatC treatment of recipient mice eliminated the conversion of serine protease zymogens to their active forms during biosynthesis, we measured the relative PR3 and NE activities in lysates of purified bone marrow-derived neutrophils using optimized FRET substrates for murine serine proteases. Enzymatic activities were determined with the substrate TAMRA-GVRRVVQYQD-dap-(CF) for murine PR3 and the ABZ-GAVVASELR-Y-(NO_2_)-D substrate for both, murine NE and PR3. We found a significant reduction of enzymatic activities in neutrophils isolated from the bone marrow of ICatC-treated mice sacrificed 4 h after transplantation. The remaining activities measured with both substrates, were clearly due to unspecific cleavage of the lysates. This was demonstrated by comparing NE and PR3 activities in neutrophil lysates from ICatC-treated mice with residual activities found in PR3/NE −/− double deficient neutrophil lysates (Fig. 1B,C). Next, we examined whether the reduced enzymatic activities after ICatC treatment correlated with the protein content of PR3 and NE. Densitometry western blot analysis of neutrophil lysates revealed no differences in the amounts of PR3 and NE antigen in vehicle and ICatC treated mice (Fig. 1D,E).

### Pre-operative inhibition of CatC improves lung oxygenation and reduces inflammatory responses

**Figure 2:**
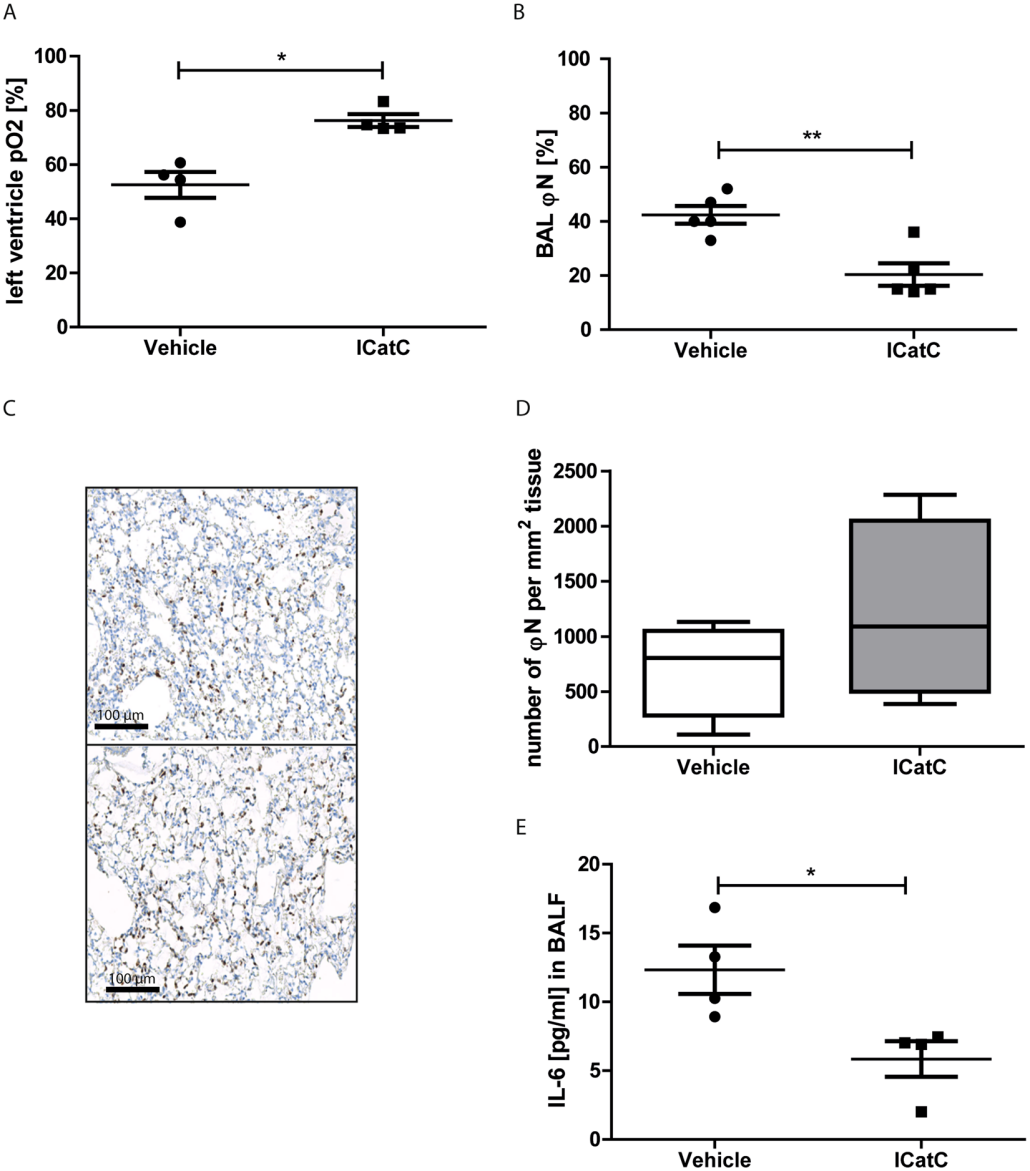
ICatC pre-treatment reduces lung dysfunction after transplantation. Lung inflammation and function was evaluated 4 hours after transplantation in C57BL/6J mice receiving either ICatC or vehicle for 10 days. (**A**) Direct measurement of the partial oxygen pressure (pO_2_) in the blood of the left ventricle after clamping the right bronchus for five minutes. (**B**) Neutrophil counts in BAL, (**C**) Immunohistochemically detection of neutrophils (Ly-6G) and (**D**) quantification of neutrophils in the transplanted left lung. Neutrophils were counted in 10 randomly chosen visual fields. *Scale bar* = 100 µm. Magnification is indicated as 20×. (**E**) BAL fluid was obtained from each group of mice and analyzed for IL-6. Statistical significance between vehicle and ICatC treatment is indicated as *horizontal brackets* and *asterisks*. Data is presented as group means ± SEM. n = 3–5 mice per group. *p ≤ 0.05 (Mann-Whitney test), **p ≤ 0.01 (independent t-test).

To determine the potential beneficial effect of pre-transplantation treatment with ICatC, we evaluated the function of the lung graft after disconnecting the blood and air supply to the non-transplanted right lung. The partial oxygen pressure (pO_2_) of the left ventricle blood in ICatC treated mice was significantly increased compared to the vehicle treated group (Fig. 2A). The content of neutrophils observed in BALF was clearly decreased in ICatC lungs compared with lungs from vehicle treated mice indicating the preservation of the capillary barrier after ICatC treatment (Fig. 2B). Neutrophil infiltration into lungs in vehicle *versus* ICatC treated mice after reperfusion injury was analyzed on paraffin-embedded sections of the transplanted lungs which were stained with the neutrophil specific Ly-6G (clone 1A8) antibody (Fig. 2C). Analytical evaluation of images showed no significant differences in the number of lung infiltrating neutrophils in both groups 4 h after reperfusion (Fig. 2D). Inflammatory mediators in BALF were analysed using a multi analyte elisa kit. IL-1A, IL-1B, IL-2, IL-4, IL-10, IL-12, IL-17A, IFNγ and TNFα were not detectable in BALF 4 h after LTx, whereas IL-6 levels were already increased at this time point. Comparing vehicle versus ICatC treated mice, we found significantly decreased IL-6 concentrations after ICatC treatment (Fig. 2E).

### PR3 and NE zymogens are present in lung tissue after ICatC treatment

**Figure 3:**
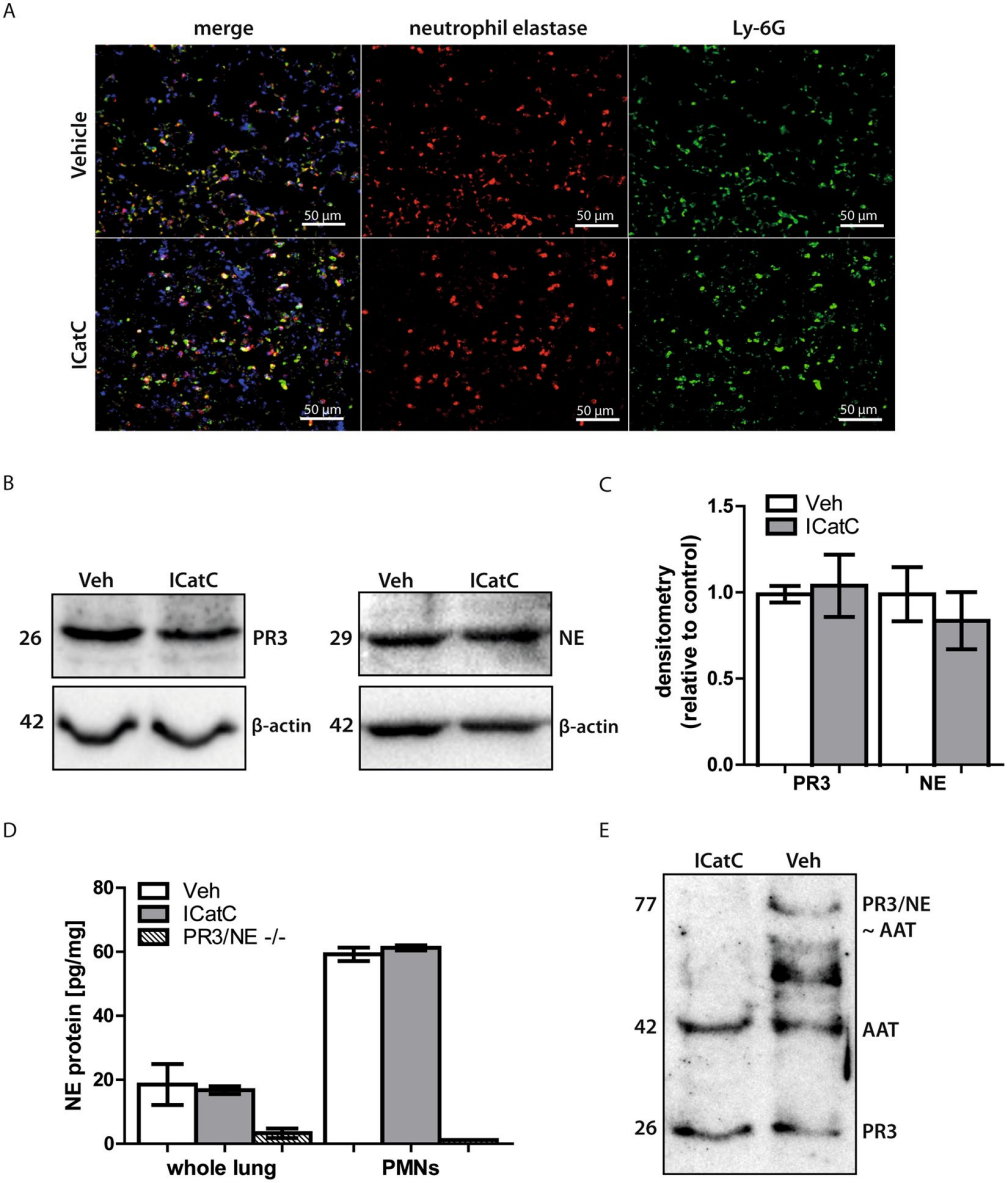
PR3 and NE zymogens are present in lung tissue after ICatC treatment. Lung tissue from vehicle and ICatC treated C57BL/6J mice was obtained 4 h after LTx. (**A**) Immunofluorescent staining of lung paraffin-embedded sections using a Ly-6G and polyclonal NE antibody (magnification 20×, scale bars = 50 µm). Sections shown are representative stainings from four vehicle and four ICatC treated mice. (**B**) Representative western blots and densitometry analysis (**C**) of whole lung lysate PR3 and NE in vehicle treated mice compared to ICatC treated mice. β-actin served as loading control (lower panel). (**D**) Quantitative determination of NE in whole lung tissue and in PMN lysates from vehicle- and ICatC-treated mice and PR3/NE double deficient (knockout) mice. Data are presented as group means ± SEM with n = 3–4 mice per group. (**E**) Simultaneous immunodetection of AAT, PR3 and AAT-complexes in whole lung tissue lysates from vehicle and ICatC treated mice. Data is presented as group means ± SEM. n = 3–5 mice per group.

Patients with germline loss-of-function mutations in both CatC alleles (*CTSC*) not only show a defect in zymogen processing, but also eliminate their zymogens before mature neutrophils are released into the blood stream^23^. In light of these data from PLS patients, we suggested that the pharmacological inhibition of CatC likewise lead to the elimination of the unprocessed pro-enzymes in mice. To investigate the fate of mouse zymogens of CatC in our experimental model after pharmacological inhibition, we stained paraffin-embedded sections of transplanted lungs with Ly-6G and NE antibodies to localize the distribution of neutrophils and NE (Fig. 3A). All neutrophils were stained positive for NE revealing the presence of the NE protein in the transplanted lung. Moreover, PR3 and NE protein levels were not altered by ICatC treatment when whole lung tissue was analyzed by semi-quantitative Western blotting (Fig. 3B,C). Full western blots are provided in supplementary information 2A,B. These findings were corroborated by quantitative measurements of the NE protein using a commercial ELISA. Indeed, we did not find significant differences in the NE levels between the experimental groups in whole lung tissues (Fig. 3D).

Since the NE antibodies used in the ELISA and Western blots were not able to distinguish between active and inactive NE it was essential to clarify whether the detected PR3 protein in whole lung lysates was enzymatically active. An AAT antibody in combination with a polyclonal anti-mouse PR3 antibody was used to detect unbound mouse PR3 and the formation of an irreversible complex with active PR3/NE, which is expected to appear as a 77 kDa band after SDS-PAGE and Western blotting. AAT is only able to form a complex with mature PR3/NE, not with the inactive pro-PR3/NE. Our results reveal that PR3/NE is not proteolytically active in the presence of ICatC as indicated by the lack of 77 kDa complex formation in comparison to vehicle treatment (Fig. 3E).

## Discussion

In this preclinical study we demonstrated that the prevention of NSP maturation in the bone marrow by CatC inhibition several days before surgery improved the quality and function of the transplanted lung immediately after surgery and could therefore represent a novel preoperative measure to increase the success of LTx in patients on the waiting list. We found that the pre-treatment of recipient mice with ICatC (1.25 mg/ml) significantly reduced the activities of the major NSPs, NE and PR3. Moreover, we provided evidence for the protective effect of ICatC on post-operative lung function and observed a significant reduction in the immediate inflammatory response after reperfusion.

In the absence of CatC activity, zymogens are synthesized in neutrophil precursors of the bone marrow, but are differentially eliminated during neutrophil maturation in mice and men. As reported for mice^9^, CatG was undetectable in bone marrow lysates, while NE protein was still present as shown by Western blotting. NE activity in CatC-deficient mice was, however, absent and residual activities were identical with background levels of NE-deficient mice^9^. These previous findings in mice with genetic CatC deficiency are consistent with our observations in CatC-inhibited mice (Fig. 1D) showing PR3 and NE proteins in lysates of purified bone marrow-derived neutrophils. These proteins were specifically located by immunofluorescence histology in graft infiltrating neutrophils after transplantation (Fig. 3A). Their identity was verified by Western blotting in total lung tissue of ICatC- and vehicle-treated graft recipients (Fig. 3B). Adding an excess of AAT, a protease activity-dependent, covalently binding inhibitor of the serpin family, we found the inactive zymogen of PR3 in ICatC-treated recipients, but active PR3 in vehicle-treated mice. Covalent complexes between serine proteases and AAT can be degraded by other proteases which most likely explain the multiple bands of smaller sizes in the vehicle control samples (Fig. 3D).

Clear evidence for the involvement of neutrophil serine proteases in ischemia-reperfusion injury and PGD development following organ transplantation has been obtained in previous pre-clinical studies^24–26^. NE and PR3 are both destructive enzymes with a wide range of substrates including various components of the extracellular matrix. In addition to matrix degradation, NE influences cell signaling by cleavage of receptors, cytokines and adhesion molecules^27^. Neutrophil extracellular traps (NETs) were recently shown to play a pathogenic role in primary graft dysfunction after lung transplantation^28^. Neutrophils that are devoid of neutrophil elastase are impaired in their formation of NETs^29^. Moreover neutrophils from CatC deficient individuals are incapable of producing NETs (18) suggesting that multiple mechanisms contribute to the beneficial anti-inflammatory effects of the CatC inhibitor in our model. The major overall effect of CatC inhibition in ischemia-reperfusion response, however, remains to be clarified.

### Clinical potential of our translational approach

Patients on the LTx waiting list usually suffer from severe end-stage pulmonary disease and need special cost-intensive care and medical supervision. End-stage lung disease often deteriorates through chronic inflammatory processes as seen with cystic fibrosis, COPD, alpha-1-antitrypsin deficiencies, and bronchiectasis. The cathepsin C inhibitor INS1007, originally developed by AstraZeneca as AZD7986, an orally applicable drug, is currently assessed in bronchiectasis patients over a 24 week treatment period (ClinicalTrials.gov Identifier: NCT03218917). In a preceding phase 1 study of healthy individuals, inhibition of CatC was well tolerated with only minor skin desquamation as side effects, but these were not considered to prevent further clinical studies^18^. CatC inhibitors have been judged to be ethically acceptable for long term application; hence they may also be considered for administration to patients on the waiting list until transplantation, at least to those with bronchiectasis. With the pre-operative ICatC treatment of the recipient it would be possible to suppress the inflammatory response initiated by the local release of NSPs from infiltrating neutrophils in the patient before transplantation and in the transplanted lungs after surgery. Based on the average neutrophil maturation kinetics in humans, treatment with a CatC inhibitor will require drug exposure for less than 3 weeks to be efficacious in humans^18,30^. This is not considered to be a limitation as transplantation candidates usually face more than 15 days of waiting for LTx. In our mouse model, 10 days of CatC inhibition were found to be sufficient to reduce serine protease activities completely. These findings are in agreement with the faster neutrophil maturation in rodents^18^.

According to a previous study, patients with a complete loss of CatC activity and affected by the PLS, normally synthesize and sort the zymogens of PR3 and NE into granules at the promyelocytic stage (18), but then eliminate these zymogens during subsequent stages of neutrophil maturation^23^ presumably because of intracellular degradation. A similar loss of zymogens as found in PLS patients was achieved through the pharmacological inhibition of CatC in a non-human primate^20^. This difference between primates and rodents with regard to the fate of neutrophil zymogens could be caused by the higher levels of zymogens and their degrading enzymes in humans or by an increased susceptibility of human zymogens to intracellular degradation. Elimination of zymogens during the maturation process in the bone marrow is a further highly welcome effect of ICatC as these zymogens might present a risk for ectopic activation by other amino-exopeptidases in the periphery. Moreover, conditions of CatC inhibition do not interfere with the recruitment of neutrophils to the site of inflammation as shown in this and a previous study in primates^20^. Hence the multiple highly redundant mechanisms of neutrophil defense responses are not impaired by ICatC treatment in general.

### Limitations of the mouse study

As the investigational novel drug INS1007 was not available to us for transplantation studies in mice, a functionally similar inhibitory substance, XPZ-01, was assessed by parental administration of two injections per day in this pilot study. Only one dose of ICatC was tested, which was chosen according to previous studies in primates and mice^20^. Many data which are routinely monitored in the clinics cannot be recorded in a small animal transplantation model under different special challenges. We have not measured blood pH, carbon dioxide in the left pulmonary artery or right ventricle and graft compliance 4 hours after left lobe transplantation. Due to the highly challenging transplantation procedure in mice we studied only the outcome of primary graft function 4 hours after LTx. Hence our data do not permit us to judge on irreversible ischemia-reperfusion injury which would have required longer observation times and additional assessment of the transplants. Our encouraging positive results, however, are a promising basis for future studies assessing beneficial effects and graft functions after longer observation and treatment periods.

In conclusion, we anticipate that pre-operative treatment with CatC inhibitors in humans is a safe and well adjustable treatment option for LTx candidates. In view of the promising phase I trials, CatC inhibitors can be rapidly translated into clinical practice. Independent of better protection of donor lungs during cold storage^8^, CatC inhibitors are a new promising drug class for the prevention of the early post-operative complications and the development of PDG in LTx.

## Methods

### Animals

Male C57BL/6J, mice were purchased from Charles River. The mice were housed with *ad libitum* access to food and water in the pathogen free animal facility of the Helmholtz Zentrum München (Munich, Germany). PR3/NE double deficient mice were established by Pfister *et al*.^31^. All animal experiments were conducted under strict governmental and international guidelines and were approved by the local government for the administrative region of Upper Bavaria (Project 55.2-1-54-2532-120-2015).

### Pre-operative ICatC treatment

10 week-old male C57BL/6L mice were treated subcutaneously for ten days twice a day with the selective CatC inhibitor, ICatC (Unizyme, Dr. Pederson; 100 µL *s.c*. per mouse, 1.25 mg/mL) or vehicle (15% (2-hydroxypropyl)-β-cyclodextrin dissolved in 50 mM citrate, pH 5)) before they underwent an orthotopic left LTx procedure.

### Murine lung transplantation model

Primary graft dysfunction (PGD) after LTx was induced as previously described^8,32,33^. 8 to 10 week-old male C57BL/6J were used as graft donors for ICatC or vehicle-treated C57BL/6J recipient mice. Donors were anesthetized with an i.p. injection of ketamine/xylazine. The pulmonary artery (PA), bronchus and pulmonary vein (PV) were carefully separated one from the other with blunted forceps, prior to cuffing with, respectively, 24G, 20G and 22G cuffs. The left lung grafts were perfused with 3 mL of Perfadex (5% dextran 40 [MW 40,000), Na^+^ 138 mmol, K^+^ 6 mmol, Mg^2+^ 0.8 mmol, Cl^−^ 142 mmol, SO_4_2^−^ 0.8 mmol, H_2_PO_4_^−^ plus HPO_4_^2−^ 0.8 mmol, glucose 5 mmol) preservation solution through the PA. PA, PV and bronchus were ligated, and the grafts were stored in Perfadex at 4C during 18 hours, to induce cold ischemia before implantation. At the time of implantation, the recipient mouse was anesthetized with a mixture of medetomidine (1 mg/kg), midazolam (0.05 mg/kg) and fentanyl (0.02 mg/kg), intubated and connected to a small animal ventilator (Harvard Apparatus), at a respiratory rate of 120 bpm and a tidal volume of 300 μl. The chest was opened on the left side between ribs 3 and 4 and the native left lung retracted with a clamp. The hilar structures were carefully separated one from the other with blunted forceps. After arrest of the blood and air flow towards the left lung, the cuffed graft PA, bronchus and PV of the donor lung were inserted into the recipient counterparts, and ligated with 9-0 sutures. The native left lung was removed and the incision in the chest was closed with a 6-0 suture, after removing all potential air bubbles from the chest. Antagonist was administrated and the animal was extubated when it showed signs of spontaneous breathing. After the operation, the recipient mice received s.c. buprenorphine (0.1 mg/kg) and were allowed to recover at 30 °C on a heating pad. The mice were sacrificed 4 hours after LTx, which is a time sufficient to induce PGD^8^.

### Donor lung function assessment

4 h after LTx, mice were anesthetized with an i.p. injection of ketamin-xylazine, intubated and mechanically ventilated with normal air, at a respiratory rate of 120 bpm and a tidal volume of 300 µl. To specifically evaluate the blood oxygenation imputable only to the left lung graft, the right bronchus was clamped, and at the same time, the tidal volume was reduced to 75 µl. After 5 min, a sample of blood was collected from the left heart ventricle. The partial oxygen pressure of this blood sample (pO_2_) was measured using a blood gas analyzer (ABL80 FLEX CO-OX Analyzer; Radiometer, Copenhagen, Denmark). Bronchoalveolar lavage fluid (BALF) was obtained from the left lung lobe by instilling 600 µl PBS while the right bronchus was still clamped. About 450 µl of BALF was obtained back and 30.000 BAL cells were used for cytospin, followed by May Grünwald (1424, Merck) and Giemsa (Merck, 9204) staining to perform a differential cell count.

### Neutrophil purification from the bone marrow

Bone marrow leukocytes were isolated from mouse femurs. After sacrificing the mouse, both femurs were dissected and bone marrow was flushed with 10 ml RPMI by using a Ø 0.04 mm syringe. Neutrophils were isolated from the bone marrow using a density gradient separation method. The bone marrow was layered over Percoll media (GE Healthcare) followed by centrifugation (500 g, 30 min, 4 °C) to separate neutrophils. Erythrocyte lysis was performed using 9 ml 0.16M NH_4_Cl and 1 ml 0.17M Tris HCl pH 7.5. Cells were washed and resuspended in phosphate-buffered saline for trypan blue cell viability assessment.

### Measurement of protease activities in cell lysates

The PR3 and NE activity in neutrophil lysates were measured fluorometrically (extinction: 485 nm, emission: 520 nm) using mouse-specific TAMRA-GVRRVVQVQD-Dap (CF) and ABZ-GAVVASELR-Y-(NO2)-D substrates (10 µM final), respectively, in 50 mM Tris, 150 mM NaCl, 0.01% Tween-20 (pH 7.4)

### Tissue preparation for histological assessment

Lung tissue biopsies were collected from the left lung lobe. The lower part of the left lung was kept at 80 °C for subsequent biological assessment and the leftover part was fixed in 4% paraformaldehyde (PFA). The fixed lung tissues were embedded in paraffin and sectioned onto slides.

### Immunohistochemical and immunofluorescent stainings

Immunohistochemical staining for neutrophil granulocytes was performed on a Discovery XT automated stainer (Ventana Medical Systems, Tucson, AZ) employing a monoclonal rat mouse-specific Ly-6G monoclonal antibody (1:200, clone 1A8, Biolegend), with Discovery Universal (Ventana Medical Systems, Tucson, AZ) as the secondary antibody. Signal detection was conducted using the peroxidase/diaminobenzidine reaction (Ventana Medical Systems, Tucson, AZ).

The stained tissue sections were scanned with an AxioScan.Z1 digital slide scanner (Zeiss, Jena, Germany) equipped with a 20× magnification objective. Images were evaluated using the commercially available image analysis software Definiens Developer XD 2 (Definiens AG, Munich, Germany) following a previously published procedure (30). The calculated parameter was the number Ly-6G postive stained cells per mm^2^ tissue.

For immunofluorescence staining, lung tissues were analyzed by a double staining for neutrophil granulocytes (1:200 monoclonal rat anti-mouse Ly-6G, clone1A8, Biolegend; 1:400 AlexaFluor ® 647 – goat anti-rat A21247, Thermo Fisher Scientific, USA) and for neutrophil elastase (1:1000 polyclonal rabbit anti-neutrophil elastase, ab68672, Abcam, UK; 1:100 Cy3-goat anti-rabbit A10520, Thermo Fisher Scientific, USA). Nuclei were identified with Hoechst 33342. Fluorescence stainings were photographed with an Axio Imager Z1 (Zeiss) and visualized with the Axio Vision 4.6.3 software (Zeiss).

### Protein analyses by Western blot and Elisa

The total protein concentration of PMN lysate, BALF and whole lungs in RIPA lysis buffer (50 mM Tris, 150 mM NaCl, 1 mM EDTA, 0.5% (w/v) deoxycholic acid, 0.1% (w/v) SDS, 0.5% (v/v) Nonidet P-40, pH 8.0) has been determined by Pierce™ BCA Protein AssayKit. BALF and lysates were separated on 12% SDS-PAGE under denaturating conditions and 10–15 µg per lane were transferred to a polyvinylidene difluoride (PVDF) membrane. Free sites on the membrane were blocked by incubation with 1× Roti^®^block (Carl Roth). Membranes were then incubated with goat anti-mouse PR3 polyclonal antibody (1:1000), rabbit anti-mNE (1:1000, ab68672, Abcam), rabbit anti-mCatG (1:1000, ab197354, Abcam) and rabbit anti-alpha 1 antitrypsin (1:1000, ab133642, Abcam) in 1× Roti^®^block at 4 °C overnight. Detection of bound primary antibodies was performed by ECL after mouse α-goat IgG (1:20000, Merck), goat α-rabbit (1:40000, SigmaAldrich), goat α-mouse IgG + IgM (1:40000, Jackson ImmunoResearch)) incubation.

Mouse elastase concentrations in whole cell lysates and IL-6 levels in BAL fluid were quantified by ELISA (PicoKine^TM^ Elisa, (EK1445), Multi Analyte ELISArray Kit (MEM-004A)) and conducted as described in the manufacturer’s recommendations.

### Statistical analysis

All results are expressed as mean ± SEM. Group comparisons were determined by student’s t-test or unpaired Mann-Whitney U-test using the commercially available statistical software GraphPad Prism version 5.0. Statistical significance was defined as p ≤ 0.05.

## Supplementary information

Supplementary Info

## Data Availability

All data generated and analyzed during the current study is available from the corresponding author on request.

## References

[1] Christie JD (2005). The effect of primary graft dysfunction on survival after lung transplantation. Am J Respir Crit Care Med.

[2] King RC (2000). Reperfusion injury significantly impacts clinical outcome after pulmonary transplantation. Ann Thorac Surg.

[3] Christie JD (2005). Impact of primary graft failure on outcomes following lung transplantation. Chest.

[4] Lee JC, Christie JD, Keshavjee S (2010). Primary graft dysfunction: definition, risk factors, short- and long-term outcomes. Semin Respir Crit Care Med.

[5] Meyer KC (2001). Neutrophils, unopposed neutrophil elastase, and alpha1-antiprotease defenses following human lung transplantation. Am J Respir Crit Care Med.

[6] Schofield ZV, Woodruff TM, Halai R, Wu MC, Cooper MA (2013). Neutrophils–a key component of ischemia-reperfusion injury. Shock.

[7] Eppinger MJ, Jones ML, Deeb GM, Bolling SF, Ward PA (1995). Pattern of injury and the role of neutrophils in reperfusion injury of rat lung. J Surg Res.

[8] Götzfried Jessica, Smirnova Natalia F., Morrone Carmela, Korkmaz Brice, Yildirim Ali Önder, Eickelberg Oliver, Jenne Dieter E. (2018). Preservation with α1-antitrypsin improves primary graft function of murine lung transplants. The Journal of Heart and Lung Transplantation.

[9] Adkison AM, Raptis SZ, Kelley DG, Pham CT (2002). Dipeptidyl peptidase I activates neutrophil-derived serine proteases and regulates the development of acute experimental arthritis. J Clin Invest.

[10] Korkmaz B, Moreau T, Gauthier F (2008). Neutrophil elastase, proteinase 3 and cathepsin G: physicochemical properties, activity and physiopathological functions. Biochimie.

[11] McGuire MJ, Lipsky PE, Thiele DL (1993). Generation of active myeloid and lymphoid granule serine proteases requires processing by the granule thiol protease dipeptidyl peptidase I. J Biol Chem.

[12] Turk D (2001). Structure of human dipeptidyl peptidase I (cathepsin C): exclusion domain added to an endopeptidase framework creates the machine for activation of granular serine proteases. EMBO J.

[13] Tran TV, Ellis KA, Kam CM, Hudig D, Powers JC (2002). Dipeptidyl peptidase I: importance of progranzyme activation sequences, other dipeptide sequences, and the N-terminal amino group of synthetic substrates for enzyme activity. Arch Biochem Biophys.

[14] Hart TC (1999). Mutations of the cathepsin C gene are responsible for Papillon-Lefevre syndrome. J Med Genet.

[15] Hattab FN, Rawashdeh MA, Yassin OM, al-Momani AS, al-Ubosi MM (1995). Papillon-Lefevre syndrome: a review of the literature and report of 4 cases. J Periodontol.

[16] Lefevre C (2001). Novel point mutations, deletions, and polymorphisms in the cathepsin C gene in nine families from Europe and North Africa with Papillon-Lefevre syndrome. J Invest Dermatol.

[17] Pham CT, Ivanovich JL, Raptis SZ, Zehnbauer B, Ley TJ (2004). Papillon-Lefevre syndrome: correlating the molecular, cellular, and clinical consequences of cathepsin C/dipeptidyl peptidase I deficiency in humans. J Immunol.

[18] Palmér Robert, Mäenpää Jukka, Jauhiainen Alexandra, Larsson Bengt, Mo John, Russell Muir, Root James, Prothon Susanne, Chialda Ligia, Forte Pablo, Egelrud Torbjörn, Stenvall Kristina, Gardiner Philip (2018). Dipeptidyl Peptidase 1 Inhibitor AZD7986 Induces a Sustained, Exposure-Dependent Reduction in Neutrophil Elastase Activity in Healthy Subjects. Clinical Pharmacology & Therapeutics.

[19] Miller BE (2017). Epithelial desquamation observed in a phase I study of an oral cathepsin C inhibitor (GSK2793660). Br J Clin Pharmacol.

[20] Guarino C (2017). Prolonged pharmacological inhibition of cathepsin C results in elimination of neutrophil serine proteases. Biochem Pharmacol.

[21] Korkmaz B (2018). Therapeutic targeting of cathepsin C: from pathophysiology to treatment. Pharmacol Ther.

[22] Gardiner P (2016). Neutrophil maturation rate determines the effects of dipeptidyl peptidase 1 inhibition on neutrophil serine protease activity. Br J Pharmacol.

[23] Sorensen OE (2014). Papillon-Lefevre syndrome patient reveals species-dependent requirements for neutrophil defenses. J Clin Invest.

[24] Fujimura N (2013). Neutrophil elastase inhibitor improves survival rate after ischemia reperfusion injury caused by supravisceral aortic clamping in rats. J Surg Res.

[25] Uchida Y, Freitas MC, Zhao D, Busuttil RW, Kupiec-Weglinski JW (2010). The protective function of neutrophil elastase inhibitor in liver ischemia/reperfusion injury. Transplantation.

[26] Harada M (2015). A neutrophil elastase inhibitor improves lung function during *ex vivo* lung perfusion. Gen Thorac Cardiovasc Surg.

[27] Pham CT (2006). Neutrophil serine proteases: specific regulators of inflammation. Nat Rev Immunol.

[28] Sayah DM (2015). Neutrophil extracellular traps are pathogenic in primary graft dysfunction after lung transplantation. Am J Respir Crit Care Med.

[29] Papayannopoulos V, Metzler KD, Hakkim A, Zychlinsky A (2010). Neutrophil elastase and myeloperoxidase regulate the formation of neutrophil extracellular traps. J Cell Biol.

[30] Summers C (2010). Neutrophil kinetics in health and disease. Trends Immunol.

[31] Pfister H (2004). Antineutrophil cytoplasmic autoantibodies against the murine homolog of proteinase 3 (Wegener autoantigen) are pathogenic *in vivo*. Blood.

[32] Krupnick AS (2009). Orthotopic mouse lung transplantation as experimental methodology to study transplant and tumor biology. Nat Protoc.

[33] Jungraithmayr W, Weder W (2012). The Technique of Orthotopic Mouse Lung Transplantation as a Movie - Improved Learning by Visualization. Am J Transplant.

