# Supplementary material for: Premedication with a cathepsin C inhibitor alleviates early primary graft dysfunction in mouse recipients after lung transplantation
Source: Sci Rep. 2019 Jul 9;9:9925. doi: 10.1038/s41598-019-46206-8 (PMC6616352; doi:10.1038/s41598-019-46206-8)
Supplement: Supplementary file 1 — Supplementary Info [file 41598_2019_46206_MOESM1_ESM.pdf]

Supplementary information

Premedication with a cathepsin C inhibitor alleviates early primary graft dysfunction in mouse recipients after lung transplantation

Salome R.T. Rehm,[1] Natalia F. Smirnova,[1] Carmela Morrone,[1] Jessica Götzfried,[1] Annette Feuchtinger,[2] John Pedersen,[3] Brice Korkmaz,[4] Ali Önder Yildirim,[1,5] and Dieter E. Jenne[1,5,6]

Figure 1

A

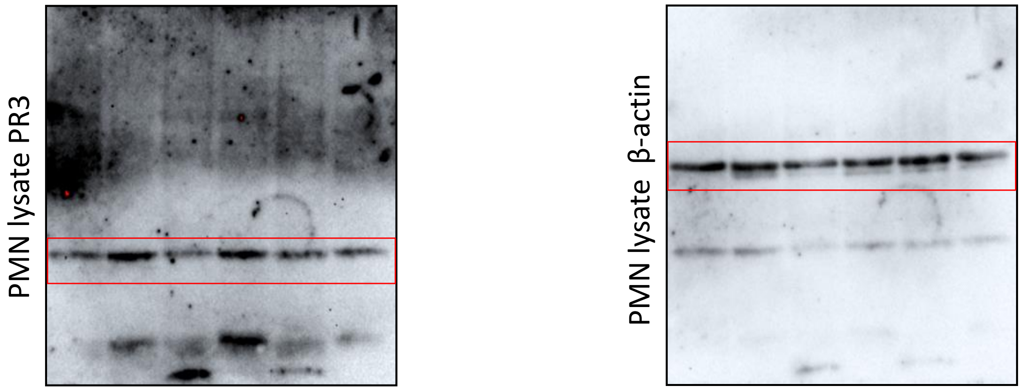

B

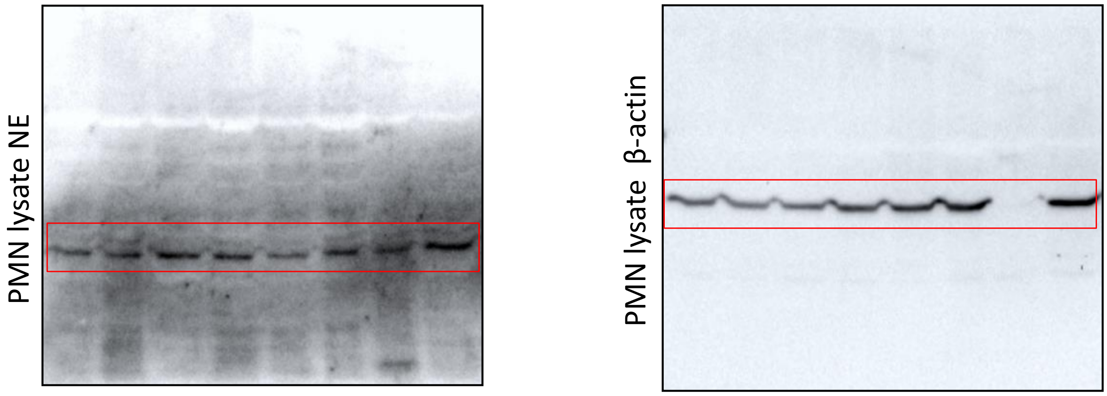

Figure 1: **Full blots shown in Figure 1D**: Blotted PMN lysates of ICatC and Veh treated mice. ICatC and Veh lysates were alternated loaded, starting with Veh. Left: (A) PR3, (B) NE. Right: Belonging β-actin signal

Figure 2

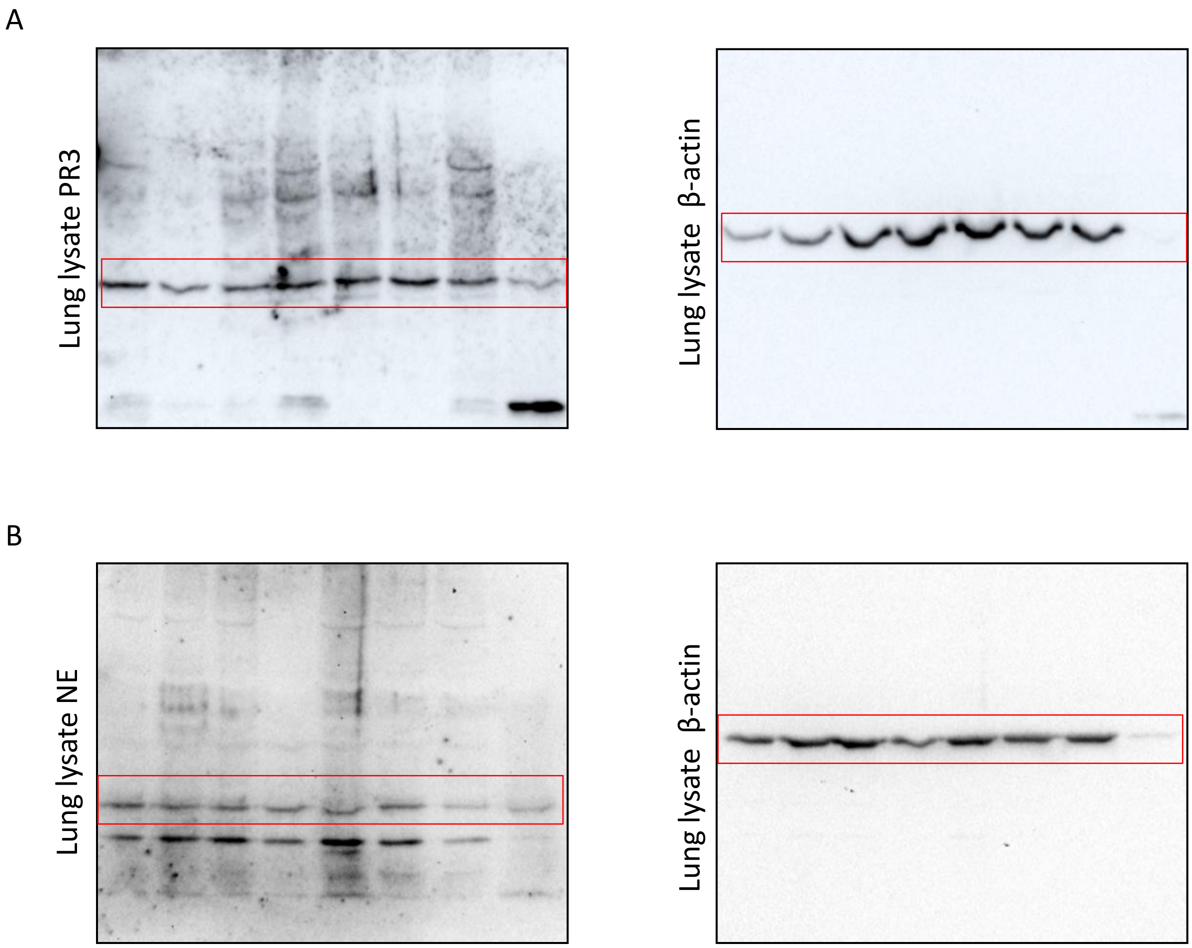

Figure 2: **Full blots shown in Figure 3B**: Blotted whole lung lysates of ICatC and Veh treated mice. . ICatC and Veh lysates were alternated loaded, starting with ICatC Left: (A) PR3, (B) NE. Right: Belonging β-actin signal
